# Supplementary material for: Clinical Characteristics and Predictors of All-Cause Mortality in Patients with Hypertensive Urgency at an Emergency Department
Source: J Clin Med. 2021 Sep 22;10(19):4314. doi: 10.3390/jcm10194314 (PMC8509826; doi:10.3390/jcm10194314)
Supplement: Supplementary file 1 [file jcm-10-04314-s001.zip › jcm-1363435-supplementary.pdf]

Supplementary Table 1. Predictors for 1-year all-cause mortality

Variables	Univariate		Multivariate	
	HR (95% CI)	*p*-value	Adjusted HR (95% CI)	*p*-value
Age (vs. < 40 years)				
40 to 59 years	2.11 (1.93–2.32)	< 0.001	3.16 (0.73–13.77)	0.125
≥ 60 years	8.18 (5.77–11.58)	< 0.001	18.89 (4.66–76.49)	< 0.001
Male sex	1.22 (0.97–1.53)	0.087	1.44 (1.07–1.95)	0.017
SBP (per 1 mmHg)	1.01 (1.01–1.02)	< 0.001		
History of hypertension	1.89 (1.49–2.39)	< 0.001		
History of diabetes mellitus	2.08 (1.64–2.63)	< 0.001		
History of ischemic stroke	2.96 (2.17–4.05)	< 0.001		
History of hemorrhagic stroke	2.01 (1.15–3.51)	0.014		
History of coronary artery disease	1.52 (1.03–2.24)	0.037		
History of chronic kidney disease	3.11 (2.27–4.25)	< 0.001		
Creatinine (per 1 mg/dL)	1.10 (1.04–1.16)	0.002		

Proteinuria	2.83 (2.11–3.81)	< 0.001	1.89 (1.38–2.59)	< 0.001
Cardiomegaly on chest radiography	2.05 (1.54–2.73)	< 0.001		
LVH on ECG	1.29 (0.89–1.88)	0.178		

HR, hazard ratio; CI, confidence interval; SBP, systolic blood pressure; LVH, left ventricular hypertrophy; ECG, electrocardiography.
